# Blood-based biomarker in Parkinson’s disease: potential for future applications in clinical research and practice

**DOI:** 10.1007/s00702-022-02498-1

**Published:** 2022-04-15

**Authors:** Lars Tönges, Carsten Buhmann, Stephan Klebe, Jochen Klucken, Eun Hae Kwon, Thomas Müller, David J. Pedrosa, Nils Schröter, Peter Riederer, Paul Lingor

**Affiliations:** 1 Department of Neurology, Ruhr-University Bochum, St. Josef Hospital, Gudrunstr. 56, 44791 Bochum, Germany; 2 Center for Protein Diagnostics (ProDi), Ruhr University Bochum, 44801 Bochum, Nordrhein-Westfalen Germany; 3 Department of Neurology, University Medical Center Hamburg-Eppendorf, 20246 Hamburg, Germany; 4 Department of Neurology, University Hospital Essen, 45147 Essen, Germany; 5 Department of Digital Medicine, University Luxembourg, LCSB, L-4367 Belval, Luxembourg; 6 Digital Medicine Research Group, Luxembourg Institute of Health, L-1445 Strassen, Luxembourg; 7 Centre Hospitalier de Luxembourg, Digital Medicine Research Clinic, L-1210 Luxembourg, Luxembourg; 8 Department of Neurology, St. Joseph Hospital Berlin-Weissensee, 13088 Berlin, Germany; 9 Department of Neurology, Universitätsklinikum Gießen and Marburg, Marburg Site, 35043 Marburg, Germany; 10 Center of Mind, Brain and Behaviour (CMBB), Philipps-Universität Marburg, 35043 Marburg, Germany; 11 Department of Neurology and Clinical Neuroscience, University of Freiburg, 79106 Freiburg, Germany; 12 Psychosomatics and Psychotherapy, University Hospital Wuerzburg, Clinic and Policlinic for Psychiatry, 97080 Wuerzburg, Germany; 13 University of Southern Denmark Odense, 5000 Odense, Denmark; 14 School of Medicine, Klinikum Rechts Der Isar, Department of Neurology, Technical University of Munich, 81675 München, Germany

**Keywords:** Biomarker, Parkinson’s disease, Parkinsonian syndromes, Alpha-synuclein

## Abstract

The clinical presentation of Parkinson’s disease (PD) is both complex and heterogeneous, and its precise classification often requires an intensive work-up. The differential diagnosis, assessment of disease progression, evaluation of therapeutic responses, or identification of PD subtypes frequently remains uncertain from a clinical point of view. Various tissue- and fluid-based biomarkers are currently being investigated to improve the description of PD. From a clinician's perspective, signatures from blood that are relatively easy to obtain would have great potential for use in clinical practice if they fulfill the necessary requirements as PD biomarker. In this review article, we summarize the knowledge on blood-based PD biomarkers and present both a researcher’s and a clinician’s perspective on recent developments and potential future applications.

## Introduction

Parkinson’s disease (PD) is a complex and heterogeneous neurodegenerative movement disorder that presents in various clinical phenotypes ranging from tremor-predominant syndromes to bradykinetic-rigid manifestations and intermediate types (Bloem et al. 2021). Similarly, disease-causing and disease-promoting pathomechanisms may be diverse and involve various molecular and cellular aspects including alpha-Synuclein (aSyn) conformational changes and its propagation, alterations in biology of amyloid-beta and tau proteins, cellular damage resulting in increase of neurofilaments, but also modified small non-coding RNA levels, lymphocyte, and inflammatory profiles.

However, these underlying processes do not consistently translate to individual clinical presentations and the individual course of disease might be quite different. Recent data from large cohort studies support the finding that mild motor-predominant, intermediate, or diffuse malignant PD progression subtypes exist (Pablo-Fernández et al. 2019), but correlations of molecular and cellular alterations with different subtypes have so far only been rare (Mestre et al. 2021). To improve the diagnosis of PD, assess disease progression, evaluate therapeutic responses, or identify PD subtypes, various tissue- and fluid-based biomarkers are currently being investigated. Blood biomarkers that are relatively easy to obtain would have a great potential for use if they were able to meet the necessary requirements in a research setting and clinical practice. In this review article, we assess the current state of blood-based PD biomarkers, and present both a researcher's and a clinician's perspective on recent developments.

## What is a biomarker?

A biomarker, as defined as a “biological marker”, is “a characteristic that is objectively measured and evaluated as an indicator of normal biological processes, pathogenic processes or pharmacological responses to a therapeutic intervention” (Biomarkers Definitions Working Group 2001, FDA-NIH Biomarker Working Group 2016). As such, a biomarker should help us (1) to understand the physiology and the pathological mechanism underlying the disease process, (2) to support the diagnostic workup including disease prediction and monitoring, and (3) to generate evidence for the therapeutic efficiency of any given treatment (Hampel et al. 2010; Jack et al. 2013).

A variety of quality criteria definitions exist for biomarker in general depending on the different application areas described above (Editorial 2010). To meet endophenotype criteria, candidate markers have to be (1) heritable, (2) relatively state-independent and stable over time, (3) associated with the illness, and (4) to be found in affected as well as unaffected family members at a higher rate than in the general population. Biomarkers should support/replace clinical endpoints, because they (1) are earlier/easier/quicker to measure, (2) reduce trial duration, size, and costs, (3) are more mechanistic, accurate, and reproducible, and (4) change dynamics in proportion to what they represent (Hampel et al. 2010; Jack et al. 2013; Ravina et al. 2005; Prasad and Hung 2021; Heldman et al. 2014; Benz et al. 2021).

Further statistical quality criteria that have to be evaluated before acceptance as a biomarker include (1) > 85% sensitivity (100% indicates that all patients are identified with the disease), (2) > 85% specificity (100% of a test identifies all individuals free of the disease), (3) positive predictive value (> 80%; refers to % of people, who are positive for the biomarker and have definite disease at autopsy), (4) negative predictive value (is the % of people with a negative test; means no disease at autopsy), and (5) prior probability (the background prevalence of the disease in the population tested) (Shaw et al. 2007; Gerlach et al. 2012).

Traditionally, biomarkers were derived as lab-based biological measures from specimens including body fluids or tissues. They are commonly analyzed with biochemical, molecular, cellular, and histological analytics or related methods derived from procedures of clinical chemistry and laboratory medicine. The present manuscript will address blood-based biomarkers, since they are the best-studied biomarkers in PD and easiest to be translated into everyday clinical practice. Particularly with respect to the relationship to clinical care procedures and implementation in clinical care, it is important to place the traditional laboratory-based biomarkers in the context of other relevant measures that could also be understood as biomarkers: clinical assessments such as quantitative scores and scales may also be considered biomarkers of a “clinical nature”, because they are widely used for the purpose originally defined for biomarkers, e.g., to measure and assess disease mechanisms and diagnostic/therapeutic procedures.

The clinical assessments are nowadays electronically recorded as electronic clinical outcomes assessments (eCOA) including clinician, observer, or patient-reported outcomes (PROMS) (Boyle et al. 2021). The ability to electronically record clinical outcomes makes these measures “available” similar to the classical biosampling procedures for lab-based biomarker. Vice versa, lab-based biomarker measures increasingly turn into data entries stored in large multidimensional registries for data-driven research approaches.

In addition, “technical biomarkers” are typically understood as biological information recorded by technical devices such as imaging technologies or objective technical measures (e.g., ECG, blood pressure, etc.). Their assessment procedure fits very well with the newly developing group of “digital” biomarker covering patient-centric technical measures such as wearable sensors and smartphone-based patient-reported outcome measures—partially referred to as “real-world outcome measures” (Rovini et al. 2017). The current biomarker developments primarily serve such purposes, as summarized in Table 1.

**Table 1:** Purposes of biomarker

Biomarker class	Application type
Diagnostic biomarker	For differential or presymptomatic diagnosis, screening for susceptibility and risks
Prognostic biomarker	For the prognosis/chance of healing, therapeutic decision support
Monitoring biomarker	For objective confirmation of therapeutic efficacy, detection of non-responsiveness, progression detection and classification, safety and quality control
Individualization biomarker	To stratify the individual patient profile to assign the patient characteristics to a better—more personalized—reference group

## Biomarker applications in Parkinson’s disease

The most extensive biomarker research for PD stems from lab-based biological measures so far. To date, however, not a single marker has been generally accepted or entered clinical practice. A major unmet need is to identify biomarkers facilitating diagnosis and therapy of PD: particularly at early disease stages, diagnosing PD can be challenging, but even at later stages, the rate of incorrect diagnoses is still remarkably high (Rizzo et al. 2016). A biomarker that is able to validate the diagnosis of PD could be helpful here. At the same time, such a diagnostic marker should be able to differentiate between idiopathic PD and other Parkinsonian syndromes (PS), such as atypical PS (aPS) and secondary PS. An example of a clinically useful and widely employed technical biomarker is the 123I-FP-CIT SPECT, which differentiates PD from other movement disorders without dopaminergic deficit with high sensitivity and specificity (Varrone and Halldin 2010). However, it fails at differentiating PD from aPS and thus remains a more general marker for identifying degenerative disorders with striatal dopaminergic deficiency. Table 2 provides an overview of currently investigated blood biomarkers for PD.

**Table 2:** Overview of blood biomarkers for Parkinson’s disease

Diagnostic biomarker	Prognostic and monitoring biomarker	Individualizing and subtyping biomarker
t-aSyn: ↑(Ng et al. 2019), ↔ (Foulds et al. 2013)) ↓(Besong-Agbo et al. 2013) in PD vs. HC	Plasma t-aSyn↑ increases over time (Foulds et al. 2013)	
Plasma exosomal aSyn↑ in PD vs. HC (Shi et al. 2014)	Plasma t-aSyn↑ predicts cognitive decline (Wang et al. 2018; Lin et al. 2017)	
o-aSyn↑ in plasma (El-Agnaf et al. 2006), serum (Williams et al. 2016), RBCs (Wang et al. 2015) in PD vs. HC		
Plasma p-aSyn↑ in PD vs HC (Foulds et al. 2013)		
Blood NfL↑ in advanced PD stages vs HC (Hansson et al. 2017; Lin et al. 2019) Blood NfL↑ in atypical parkinsonian syndromes (APS) vs PD (Lin et al. 2019; Hansson et al. 2017; Marques et al. 2019)	Blood NFL↑ is associated with more severe motor impairment (Ygland Rödström et al. 2021; Ye et al. 2021) and cognitive decline (Choe et al. 2020; Niemann et al. 2021; Lin et al. 2019; Buhmann et al. 2022; Aamodt et al. 2021; Ma et al. 2021) over time	Blood NFL↑ in PD-PIGD vs PD-TD (Ng et al. 2020)
Aß42: (Chojdak-Łukasiewicz et al. 2020), ↔ (Lin et al. 2018a), ↓ (Ding et al., 2017) in PD vs. HC		Plasma Aß42↓ in PIGD vs. TD subtype in PD (Ding et al. 2017)
Plasma t-tau↑ (Chen et al. 2020) and serum t-tau ↔ (Chojdak-Łukasiewicz et al. 2020) in PD vs HC	Plasma t-tau↑ correlates with worse cognitive function (Chen et al. 2020; Lin et al. 2022)	
Plasma p-tau↑↑ x Aß42↑ in FTD vs PD/APS (Lin et al. 2018a)	Plasma EV Aß42↑ and t-tau↑ predict cognitive decline (Chung et al. 2021)	
Plasma IL-6, IL-10 ↑ (Qin et al. 2016) PD vs HC		Il-6 ↑ predicts depression (Veselý et al. 2018)
		Il-10 ↑ correlates with gastrointestinal impairment, anxiety and depression (Karpenko et al. 2018; Kim et al. 2018)
Whole blood/Serum CRP↑ (Qiu et al. 2019) PD vs HC		CRP ↑ associated with reduced survival (Sawada et al. 2015)
		Proinflammatory cytokine ↑ anti-inflammatory cytokine ↓ are associated with worse motor and cognitive impairment (Williams‐Gray et al. 2016)
↑ in PD vs. HC: miR-6836-3p, miR-6777-3p ↓ in PD vs. HC: miR-493-5p, miR-487b-3p, miR-15b-5p (Kern et al. 2021)	71 miRNAs identified to correlate with disease progression in PD (Kern et al. 2021)	

*aSyn* alpha-synuclein, *t-aSyn* total alpha-synuclein, *o-aSyn* oligomeric alpha-synuclein, *p-aSyn* phosphorylated aSyn, *RBC* red blood cell, *Aß40* amyloid-β peptide 42, *Aß42* amyloid-ß peptide 40, *t-tau* total-tau, *p-tau* phosphorylated tau, *FTD* frontotemporal dementia, *APS* atypical Parkinsonian syndromes, *EV* extracellular vesicles, *PD* Parkinson’s disease, *HC* healthy controls, *NLR* Neutrophil-to-Lymphocyte ratio, *Tregs* T regulatory cells, *IL* interleukin, *TN* tumor necrosis factor, *IFN* interferon, *DaTSCAN SBR* striatal binding ratios in Dopamine Transporter Single Photon Emission Computed Tomography, *CRP* C-reactive protein, *PIGD* postural instability gait difficulty, *TD* tremor-dominant

Biomarkers could also facilitate clinical trials as surrogates for disease progression. Whereas in clinical routine, disease progression is mostly quantified by an increase in a symptom severity score and application of clinical-type biomarker (e.g., MDS-UPDRS, NMSS, or PDQ-39), symptom severity scores can be significantly skewed by symptomatic therapies that are commonly used in PD. A laboratory-based biomarker that indicates disease progression independent of clinical symptoms could be very useful, particularly for clinical trials of disease-modifying substances. For example, procalcitonin is a widely used biomarker for sepsis and indicates therapeutic response well before the patients’ clinical condition improves (Carrol et al. 2002).

Finally, biomarkers could facilitate the identification of subtypes of patients with similar clinical phenotypes, but differing etiopathogenesis. For example, genotyping can identify multiple independent genotypes that are responsible for the development of a PD phenotype. Clearly, also idiopathic PD is a heterogeneous disorder, even in prodromal stages (Berg et al. 2021; Qian and Huang 2019). Biomarkers permitting the identification of defined molecular subgroups could enable the selection of clinical subgroups for clinical trials and subsequently pave the way for tailored therapies. Such molecular stratification strategies are already successfully employed in clinical oncology, where, for example, breast cancer subtypes expressing certain hormone and/or growth factor receptors have direct implications for therapeutic decisions (Bou-Dargham et al. 2021).

The following section reviews the most extensively studied and best characterized blood-derived laboratory-based biomarkers for PD concerning (differential) diagnosis, disease progression/therapy response, and PD subtype differentiation/individualization.

## Blood-based biomarker

### Alpha-synuclein

Alpha-synuclein (aSyn) is a small protein of 140 amino acids located at the presynaptic nerve terminals where it presumably exerts its regulatory role in synaptic function and neurotransmitter release (Stefanis 2012). Importantly, this protein is neuropathologically and genetically closely linked to PD. aSyn undergoes extensive posttranslational modifications including phosphorylation as well as conformational transformations leading to the formation of aggregates and neuronal toxicity (Zhang et al. 2019). Since aSyn is considered central to PD pathogenesis, it is most promising to serve as a biomarker.

Beyond its neuronal expression, aSyn can also be detected in blood, most abundantly in erythrocytes (red blood cells; RBCs) (Barbour et al. 2008). However, studies on total aSyn revealed inconsistent results. As compared to controls, total aSyn levels in PD serum or plasma were either increased (Lin et al. 2017; Ng et al. 2019), not significantly different (Mata et al. 2010; Goldman et al. 2018), or even reduced (Li et al. 2007; Besong-Agbo et al. 2013). Preanalytical factors, particularly aSyn contamination from RBCs due to hemolysis, use of different analytical assays, and sampling procedures across studies, may have contributed to these discrepancies. Notably, higher aSyn levels in plasma and serum showed a significant correlation with motor severity (Wang et al. 2019a; Chang et al. 2020) and cognitive decline (Lin et al. 2017; Ng et al. 2019). Exosomes are part of extracellular vesicles (EV) that can transfer αSyn across cells and from brain to the peripheral blood. Significantly higher concentrations of exosomal aSyn have been observed in PD patients compared to controls (Shi et al. 2014). A recent study with a small sample size and a follow-up period of 22 months confirmed this finding in patients with early stage PD, although not baseline, but longitudinal increase of exosomal aSyn level was associated with higher risk of motor progression (Niu et al. 2020).

Given the conflicting data on total aSyn levels, attention has been directed to more disease-specific types of aSyn. Oligomeres of aSyn are considered as toxic forms that precede aggregation and neuronal death. Increased levels of oligomeric forms of aSyn have been found in plasma (El-Agnaf et al. 2006), serum (Williams et al. 2016), and RBCs (Wang et al. 2015; Daniele et al. 2018) with moderate ability to differentiate PD from controls. Phosphorylation at serine 129 is the most common posttranslational modification of aSyn. PD patients displayed elevated levels of phosphorylated aSyn in plasma (Foulds et al. 2013) and in RBCs (Tian et al. 2019). The diagnostic accuracy can be increased by composite biomarkers including total, proteinase-K resistant, and phosphorylated aSyn that differentiated PD from controls with an AUC of 0.81 (Abd Elhadi et al. 2019). One of the rare longitudinal studies assayed total aSyn and phosphorylated aSyn at 4–6 monthly intervals from plasma of 189 newly diagnosed PD patients over a period of 3–4 years (Foulds et al. 2013). Whereas total plasma aSyn levels in PD were not different from controls at baseline and increased over time, higher phosphorylated aSyn levels were found in PD plasma which remained stable during the follow-ups. Regarding the total aSyn dynamics, a higher clearance from the site of aSyn pathology to the general blood circulation has been discussed. Another longitudinal study reported that both baseline and longitudinal increases in total plasma aSyn predicted the progression of cognitive decline in individuals at risk for PD, presenting with hyposmia and dopamine transporter (DAT) binding reduction (Wang et al. 2018).

Overall, we conclude that the plasma level of oligomeric and phosphorylated aSyn has potential value as a diagnostic tool, whereas the level of total aSyn could act as a surrogate marker for the progression of PD. The utility of aSyn containing exosomes needs to be further validated. Standardization of preanalytical factors to allow comparability will be necessary.

### Amyloid-beta/tau

Amyloid beta peptides (Aβ) are cleaved from the amyloid precursor protein (APP) into the isoforms Aβ42 and Aβ40 that can further assemble into extracellular plaques (Chen et al. 2017). Hyperphosphorylation of tau protein can lead to intracellular accumulations appearing as neurofibrillary tangles (NFTs) (Lei et al. 2010). Amyloid plaques and NFTs are characteristic of Alzheimer’s disease (AD), but can also coexist with Lewy body pathology in PD correlating with an accelerated cognitive decline (Jellinger et al. 2002; Irwin et al. 2013). Aβ and tau synergistically interact with aSyn promoting their mutual accumulation (Clinton et al. 2010).

In CSF, a trend toward lower Aβ42 levels has been reported in PD, mostly associated with cognitive dysfunction (Alves et al. 2014; Parnetti et al. 2014). Aβ42 can also be measured in blood. Earlier reports did not find a correlation with cerebral Aβ pathology as indicated by CSF or positron emission tomography (PET) (Zetterberg 2015). It has been discussed that high amounts of the Aβ peptides in plasma could have derived from extra-cerebral sources such as platelets leading to these negative findings (Lim et al. 2019). However, novel ultrasensitive immunoassay technologies including immunomagnetic reduction (IMR) suggest that plasma Aβ level could correlate with cerebral amyloidosis (Teunissen et al. 2018). Only a few studies have assessed Aβ42 in PD blood, reporting inconsistent findings regarding blood level or correlation with cognitive functions (Lin et al. 2018a; Chojdak-Łukasiewicz et al. 2020; Chen et al. 2020). With regard to PD motor symptoms, lower Aß42 levels in plasma were shown to be associated with postural instability gait difficulty (PIGD) subtype (Ding et al. 2017). Of note, this motor subtype in turn predisposes to the risk of developing cognitive impairment.

Evidence on tau protein level in PD blood is even more limited. Furthermore, rapid changes in tau concentrations in blood as demonstrated in patients with acute hypoxic brain injury could hamper a correlation with CSF tau (Randall et al. 2013). Increased and similar total tau levels have been observed in PD compared to controls (Chen et al. 2020; Chojdak-Łukasiewicz et al. 2020). A recent study with 55 PD patients did not compare total tau levels with controls, but demonstrated a correlation between higher t-tau levels with lower cognitive performance (Lin et al. 2022). Among patients with parkinsonian syndromes, plasma levels of total and phosphorylated Tau were significantly increased in all disease groups compared to controls, with the highest level in patients with FTD that in combination with Aβ42 could differentiate FTD from PD/APS with high accuracy (Lin et al. 2018a).

Aβ and tau have also been detected in exosomes/extracellular vesicles (EV). Although tau and Aß42 plasma EV levels did not differ between PD patients and controls, elevated levels of both proteins were significantly associated with cognitive impairment and combined with other parameters including aSyn in EV identified cognitively impaired PD patients with high accuracy (Chung et al. 2021).

Taken together, given the limited number of studies that primarily had a cross-sectional design and only small sample size, it remains unclear to what extent AD core peptides in the blood can reflect neuronal conditions in PD. Larger studies using ultrasensitive assay methods are needed to better assess the validity of Aβ and tau particularly for detecting and tracking cognitive dysfunction in PD. Future investigations should also include Aβ42/Aβ40 ratios to correct for interindividual differences.

### Neurofilament light chain

Neurofilament light chain (NfL) is a structural protein highly expressed in axons and released upon neuronal damage, rendering it a robust marker for neuronal injury. NfL concentrations in blood and CSF strongly correlate (Hansson et al. 2017; Wang et al. 2019b; Marques et al. 2019). Therefore, blood NfL is a promising biomarker for neurodegeneration including Parkinson´s disease (PD). CSF NfL levels in different Parkinsonian syndromes were found to correlate with pathological changes in presynaptic putaminal dopamine transporter (DAT) density reflecting nigrostriatal degeneration (Diekämper et al. 2021) and higher baseline serum NfL was associated with greater reduction of putaminal DAT-binding ratio over time (Ye et al. 2021).

Blood NfL is higher in atypical parkinsonian syndromes (APS), such as multiple system atrophy (MSA), progressive supranuclear palsy (PSP), and corticobasal syndrome (CBS) (Lin et al. 2019; Hansson et al. 2017; Marques et al. 2019; Mollenhauer et al. 2020) compared to PD and therefore might serve as biomarker to distinguish between APS and PD, even in early stages of APS, when clinical symptoms are not yet conclusive (Hansson et al. 2017; Marques et al. 2019). Accuracy levels up to 91% (sensitivity = 86% and specificity = 85%) for distinguishing APS from PD applying a cut-off value of 14.8 g/L have been suggested (Marques et al. 2019). In PD and controls, but not in APS (Marques et al. 2019), serum NfL concentrations correlate with age (Lin et al. 2018b, 2019; Marques et al. 2019; Mollenhauer et al. 2020).

Blood NfL seems to be higher in more advanced PD patients compared to controls, while the situation is described controversially for early disease stages. A metaanalysis showed no differences in blood NfL in PD patients not stratified by disease severity compared to controls (Wang et al. 2019b). While recently blood NfL concentrations at baseline and at follow-up (mean of 6.4 years) were found higher in de novo PD patients compared to controls (Ma et al. 2021), others did not find a difference for earlier PD disease stages (means for age, H&Y, and disease duration: 57y/2.0/34mo) (Marques et al. 2019). In another study, only in advanced PD patients (65y/2.5/9.7y) but not in a less severely affected group of comparable age (65y/1.9/5.3y), NfL blood levels were higher compared to controls (Hansson et al. 2017). For advanced PD patients (68.5y/3.1/7.8y), a plasma NfL cut-off value of 12.34 pg/ml has been suggested to have a modest sensitivity (53.2%) and a high specificity (90.5%) for distinguishing between patients and controls (Lin et al. 2019).

Cross-sectional studies showed heterogeneous results with respect to association of blood NfL concentrations with motor impairment in PD, revealing positive (Lin et al. 2019; Niemann et al. 2021) and negative results (Oosterveld et al. 2020). Higher NfL levels are associated with more severe motor impairment in the long-term disease course. Baseline serum NFL levels showed significant hazard ratios for four out of five disease progression milestones in the long-term course, indicating that patients with higher blood NFL levels have a higher likelihood for later motor and social impairment, expressed by the need for a walking-aid, nursing-home living, reaching final H and Y stage 5 or death (Ygland Rödström et al. 2021). In de novo PD patients, higher baseline serum NfL was associated with greater increases of UPDRS-III and total UPDRS scores, with greater worsening of postural instability and gait disorder (PIGD) scores—but not tremor scores—over time (Ye et al. 2021).

Blood NfL was also found associated with *PIGD-subtype* of PD (Pötter-Nerger et al. 2022; Ng et al. 2020). At 2 year follow-up, NfL levels were higher in PIGD compared to tremor-dominant subtype, and within the PIGD group, higher blood NfL was associated with worse global cognition and UPDRS III at baseline and predicted motor and cognitive decline (Ng et al. 2020). Furthermore, NfL baseline levels were associated with greater worsening of PIGD scores over 8 year follow-up (Kim and Jeon 2021).

Consistent inverse associations of blood NfL levels with cognitive scores have been reported (Oosterveld et al. 2020; Lin et al. 2019, 2018b). Except for one study (Ye et al. 2021), all identified publications described a positive association between baseline blood NfL levels and negative cognitive outcome by applying different cognitive measurement instruments. This association was found for all PD severity stages, i.e., in a heterogenous PD cohort (Aamodt et al. 2021), in prodromal and de novo patients (Mollenhauer et al. 2020; Ma et al. 2021), in moderately (Lin et al. 2019), and advanced (Choe et al. 2020; Niemann et al. 2021) diseased PD patients. NfL values in the range from 14 to 19 pg/ml have been suggested as cut-off values for a higher risk of cognitive decline (MoCA) (Aamodt et al. 2021; Lin et al. 2019), but likely cohort-related age-adjusted NfL levels are more reliable (Buhmann et al. 2022; Ma et al. 2021).

Furthermore, blood NfL and NT-proBNP levels are associated with PD (Niemann et al. 2021) indicating subclinical cardiac damage. In line, cardiac microdamage and vascular risk factors have been found associated with motor symptoms and progression in PD (Choe et al. 2020; Mollenhauer et al. 2019) which might be part of the body-first type of PD (Horsager et al. 2020).

Overall, there are increasing data suggesting blood NfL as biomarker for disease severity and predictor for cognitive decline. Blood NfL concentration seems to be associated with PD patients of the PIGD subtype and subclinical cardiomyopathy.

### sncRNAs

RNA species that are not translated into a protein, but contribute to the regulation of gene expression are called non-coding RNA (ncRNA). In contrast to long non-coding RNA, small ncRNA (sncRNA) are shorter than 200 nucleotides and consist of different species, such as miRNA, piRNA, and siRNA. SncRNA has been previously shown to have a potential as biomarker in various types of cancers (Esteller 2011). However, the identification of such markers is complicated, among other things, by the fact that blood is composed of different non-exclusive components, e.g., plasma, serum, PBMC, or exosomes, each of which contains sncRNA in a different composition (Roser et al. 2018). Furthermore, the method that is used to detect and quantify sncRNAs as well as the sample pre-analytics may significantly alter the results of sncRNA analyses (Kuo et al. 2021).

A multitude of PD patient cohorts has been analyzed for differential expression of sncRNA in the hope to identify individual sncRNAs or combinations thereof that can be used as a biomarker. One of the largest studies to date used 4.440 samples from 1.511 individuals in the discovery cohort and 1.440 samples from 988 donors in an independent validation cohort (Kern et al. 2021). As in most other studies, more than 90% of all detected sncRNA species belonged to miRNA. Analysis of the discovery cohort showed miR-6836-3p and miR-6777-3p to be upregulated in PD, whereas miR-493-5p, miR-487b-3p, and miR-15b-5p supported the general trend of miRNA downregulation in PD patients compared to controls. The latter two were also reproduced in the validation cohort. Being able to study longitudinal data, the authors also suggest that miRNAs can be used as progression markers in PD. Although the miRNA identified in this study are involved in biological processes that are commonly associated with PD pathogenesis (mitochondrial function, oxidative stress, and protein degradation), the correlation of the deregulated miRNAs with those identified in previous studies is limited. Only miR-15b-5p was previously shown to be regulated in the same direction (Ding et al. 2016). The authors clearly show an age-dependence in the expression of sncRNA and, by deconvolution, relate the origin of the miRNAs mostly to cells of the immune system. Other summaries of the recent work on blood-based sncRNA biomarker in PD display a plethora of different miRNA that were found to be deregulated in cohorts of different sizes and compositions (Schulz et al. 2019; Kuo et al. 2021; Roser et al. 2018).

Although there is no doubt that sncRNAs play an important role in the regulation of disease-relevant mechanisms and show a correlation with disease state and progression, it remains to be determined if they can be established as a clinically useful biomarker for PD. The weak correlation of individual sncRNA targets even from large studies does not allow designating specific sncRNAs as biomarker in PD. An important prerequisite for comparability between studies will be the choice of a uniform sampling source as well as a uniform quantification and analysis pipeline. Other sources which may show less variability, such as CSF, may be equally promising to explore (Caldi Gomes et al. 2021).

### Lymphocyte cell profiles

Inflammation that is associated with neurodegeneration is gaining increasing attention in PD research. However, there is an ongoing discussion on whether inflammation is an important trigger factor for disease development or a consequence of degeneration (Hirsch and Hunot 2009).

A variety of studies with mostly retrospective and cross-sectional approaches have described alterations attributable to both pro- and anti-inflammatory cellular responses in PD. ASyn was identified as a possible causal link between PD and inflammation, as it was shown to be able to trigger specific responses in both helper and cytotoxic *T* cells (Sulzer et al. 2017). T helper cells, also called CD4 + *T* cells, occupy a key position in the regulation of the immune system via cytokines and, taken together with CD8 + *T* cells, comprise the largest proportion of *T* cells (Luckheeram et al. 2012). CD4 + *T* cells can adopt both a pro- (e.g., T helper (Th) 1) and an anti-inflammatory phenotype (e.g., Th2 or T regulatory (Treg)). Regardless of dopaminergic therapy, a decline of CD4 + cells was observed with a shift toward Th1 and a concomitant decrease in Th2 and Treg, representing a tipping of the balance toward a proinflammatory state (Kustrimovic et al. 2018; Rocha et al. 2018; Sun et al. 2019). Regarding Tregs, only the subtypes of suppressor, active and type 1 Tregs are diminished in PD, whereas classic and resting Tregs remain stable (Álvarez-Luquín et al. 2019). In contrast, Schröder et al. found no difference in the total number of CD4 + and CD8 + T lymphocytes, but observed an increased proportion of activated cells (Schröder et al. 2018). In parallel, a shift from naive to activated Treg cells (CD45RA + to CD45RO +) was observed with higher levels of CD45RO + cells associated with more severe motor and cognitive impairment (Magistrelli et al. 2020; Saunders et al. 2012). The proportion of Tregs expressing CD49d, coding for a molecule that enables migration into the central nervous system, is increased in PD and linked to lower motor impairment, suggesting a protective influence (Karaaslan et al. 2021). Significantly decreased expression of CD57 was observed on CD8 + *T* cells accompanied by increased expression of CD28. This is inverse to the surface expression observed in physiological immunosenescence, indicating pathological immunological aging in PD and representing a possible link to the increasing incidence of disease with age (Williams-Gray et al. 2018).

In addition to lymphocyte profiles analyzed by flow cytometry, the neutrophil-to-lymphocyte ratio (NLR) is increasingly reported as an easily determinable and cost-effective biomarker. NLR is an indicator of the inflammatory status, that is evaluated not only in infectious but also in neurodegenerative disorders such as Alzheimer’s disease (Dong et al. 2019; Russell et al. 2019). Elevated NLR was found in PD compared to healthy controls in a case–control study and confirmed in a subsequent meta-analysis of nine studies (Muñoz‐Delgado et al. 2021). Late-onset PD is reported to have a higher NLR than early onset PD (Jin et al. 2020). Regarding paraclinical and clinical parameters, a negative association between NLR and striatal-binding ratios in DaTScan analysis as a surrogate parameter for nigrostriatal degeneration was observed as well as a positive association with motor impairment indicating NLR as a potential biomarker for disease progression. However, this is limited by the fact that the associations were observed only for tremor-dominant PD but not for other subtypes (Muñoz‐Delgado et al. 2021, Sanjari Moghaddam et al. 2018).

Taken together, lymphocyte profiles and NLR represent an interesting approach as biomarkers for both diagnosis and disease progression of PD. While the NLR is supported by its easy accessibility and association with nigrostriatal degeneration as one of the pathophysiological hallmarks of PDs, lymphocyte profiles could potentially play a role in the identification of subtypes within PDs and contribute to the pathophysiological understanding of the disease. Further prospective studies with clearly defined a priori hypotheses and sufficient power are needed to conclusively assess the value of lymphocyte profiles as biomarker.

### Cytokines and other inflammatory markers

The altered composition of lymphocytes leads to and is in turn influenced by an altered composition of cytokines. Several cytokines and inflammatory markers were investigated as a potential biomarker in PD. Elevated levels of CRP and hs-CRP were found in patients with PD compared to healthy controls in a large number of studies, though with limited sample sizes. These results have now been confirmed in recent meta-analyses (Akıl et al. 2015; Jin et al. 2020; Qiu et al. 2019). Additionally, higher CRP levels, measured in an infection-free interval, are associated with reduced survival (Sawada et al. 2015).

Interleukins comprise a group of more than 50 cytokines that can exert both a pro- and anti-inflammatory effect (Brocker et al. 2010). Results to date suggest differences in interleukin levels between PD and healthy controls, although interleukins must be considered separately, and also conflicting results have been published in recent years. For Il-6 and Il-10, a meta-analysis of 13, respectively 5, studies demonstrated increased levels compared with healthy individuals, though with only a small-to-intermediate effect size (Qin et al. 2016). Subsequent studies yielded mixed results and found no difference (Schröder et al. 2018) or even decreased interleukin levels compared to healthy subjects (Rocha et al. 2018). A positive association was observed between Il-6 and depression in a 2 year follow-up (Veselý et al. 2018). Concerning Il-10, a positive association with autonomic, especially gastrointestinal symptoms, was described, while others found a positive association with anxiety and depression (Karpenko et al. 2018; Kim et al. 2018).

Compared to interleukins, data for TNF-α are even more inconclusive with mixed results being reported for TNF-α levels, with increased (Dufek et al. 2009; Kouchaki et al. 2018; Wang et al. 2016), decreased (Gupta et al. 2016), or equal levels (Lindqvist et al. 2012) compared to healthy controls. Nevertheless, associations were observed with disease stage, fatigue, depression, and anxiety (Menza et al. 2010). Chemokine ligand 5 (CCL5, RANTES) levels were elevated in PD compared to controls with a positive correlation with Hoehn and Yahr stage, motor impairment, and disease duration (Rentzos et al. 2007; Tang et al. 2014). In a large prospective study, elevated proinflammatory and low anti-inflammatory cytokine levels were associated with an unfavorable outcome for both motor deficits and cognition (Williams‐Gray et al. 2016). However, these results could not be confirmed in a trial with a similar design (Yilmaz et al. 2018).

Taken together, the value of individual inflammatory markers in PD is low as both diagnostic and prognostic biomarker. Studies that combine several pro- and anti-inflammatory markers showed promising results both in diagnostic performance and as predictors of individual disease progression (Rathnayake et al. 2019; Williams‐Gray et al. 2016). More prospective studies that are adequately powered are required to conclusively assess the potential of inflammatory markers in PD.

## Methods for biomarker identification using machine learning techniques

Biomarker development is particularly challenging in diseases that involve multiple subgroups or when a confirmed diagnosis cannot be made until autopsy, as is the case with Parkinson’s disease. This represents a vicious circle, e.g., in PD patients with multifaceted manifestations, as the demand for universal biomarkers has often resulted in them being representative of PD subtypes at best (Eggers et al. 2012). One solution could lie in increasing computational capacity for multiparametric assessment and improving data availability, including data-driven biomarker identification based on clinical data (Xie et al. 2021; Mamoshina et al. 2018). The basis for biomarker identification using database information is a thorough clinical description and a sufficient number of samples. Databases containing information on a large number of participants have been previously established and are in part openly available to researchers (Simuni et al. 2016; Usnich et al. 2021). However, accessible data are not sufficient for biomarker development and new possibilities of data analysis in large samples have revealed limitations in traditional statistical analyses. For example, divergent clinical and statistical significance may require cautious interpretation, as do small-effect sizes with high case numbers. Moreover, rather trivial problems are encountered, such as, e.g., the handling of excessive type I errors (Smith and Nichols 2018). Yet, problems of frequentist approaches may be remedied with probabilistic methods such as Bayesian statistics or what is broadly categorized as artificial intelligence.

Thanks to ever-increasing computing power, machine learning approaches have found their way into everyday life, but especially into science. Such approaches usually train in a representative dataset and are verified in the second set of data with characteristics as similar as possible (Mitchell^ 2010^). Two approaches to biomarker discovery can be identified: classification and feature selection. For PD patients specifically, classification, on the one hand, may enable stratification according to, e.g., disease progression, age at onset, but more recently also to genetic profiles (Simuni et al. 2020); machine learning techniques thereby enable almost unlimited complexity, permitting the inclusion of thousands of genes or imaging results. Feature selection methods, otherwise, can be divided into (1) filter methods selecting features as per, e.g., correlations, (2) wrapper methods using objective functions such as classification accuracy to identify important feature combinations among all possibilities, and (3) embedded functions that incorporate feature selection and penalize overfitting (He and Yu 2010). In general terms, feature selection aims at liberating data from factors with only limited contribution, so that interesting markers remain. What sounds simple and purposeful, however, also has some disadvantages in practice.

Classical statistical inference methods are beyond doubt in terms of scientific verification and application for biomarker discovery. To what extent machine learning algorithms will enable biomarker identification remains yet unresolved, whereas some aspects are already becoming clear. In contrast to traditional statistical analyses necessitating specific distributional assumptions which may not always be met, machine learning usually requires fewer assumptions. More importantly, however, interactions between variables can be accounted for—approximating closer to complex biological systems. Particularly, combining feature selection and classification has already been put into practice (Leclercq et al. 2019). Otherwise, especially unsupervised algorithms deliver conflicting specificity and accuracy results, a major drawback. Thus, ethical and practical implications arising from that dilemma have yet to be assessed, such as the question of how to disclose the lack of explicability of the algorithms and the resulting medical decisions to patients (Beil et al. 2019) or how the fact should be handled that important clinical aspects may not have been incorporated to the training, so that generalizability may be questionable. Besides, the resulting multidimensional patterns must not be fully intuitive, as they may not be traceable or show a relationship to the original data. These findings can emerge as a sort of “black box”-like markers which, however, have scientific value and have to be put into relation with other already established biomarkers or clinical findings. To a certain degree, one may therefore argue that machine learning models and the set of features may be coined as biomarker, themselves. Finally, it needs to be borne in mind that multidimensional biomarker patterns are only tangible and useful through representative training data sets.

## Current trends in PD biomarker research

Currently, a biomarker with a high sensitivity and specificity does not exist for diagnostic and/or therapeutic purposes in PD. The heterogeneity of PD is caused by both phenotype and genotype or epigenetic factors. Especially, the results of molecular genetics over the last decades have raised hopes of earlier diagnosis and opened new therapeutic neuroprotective approaches (Cook et al. 2021). However, there is a lively debate on the etiology of the disease and some studies challenge theories that have been favored for a long time (Horsager et al. 2020; Fearon et al. 2021). For example, the “brain first versus body first” dichotomization (Horsager et al. 2020) classified PD patients into two subgroups with premotor rapid eye movement (REM) sleep behavior disorder (RBD) at least 1 year prior to the onset of a motor phenotype (“body-first”) and those characterized by the absence of RBD at motor onset (“brain-first”). However, neither is this simplification sufficient for patients in clinical practice nor for clinical studies. Therefore, while understanding the underlying disease mechanism is key, the goal of a single biomarker for PD patients will be difficult to achieve in the future. We have to move away from the idea of a single, ideal biomarker for diagnostic, progression purposes, or monitoring therapeutic interventions in PD. As mentioned above, there is a significant overlap of a potential biomarker such as aSyn species, Aß, tau, and miRNA (Sadlon et al. 2019) in many patients with neurodegenerative diseases like AD and PD.

Further research activity will be necessary to improve current or identify new techniques for the analysis of biomaterials. The real-time quaking-induced conversion (RT-QuIC) is one example of a promising new tool for measuring aggregated aSyn in CSF of PD which could be applied also to blood specimens (van Rumund et al. 2019; Iranzo et al. 2021). However, the value of RT-QuIC in diagnostics or for monitoring the disease course is controversial (Nakagaki et al. 2021; van Rumund et al. 2019). In addition, pattern recognition methods will play a major role in the identification of disease pathways, as has been previously shown in cancer research (Cheng and Zhan 2017). The selection of cohorts included in biomarker analyses is also of utmost importance and needs to be considered when interpreting data from biomarker trials. Findings from other fields may lead the way: in analogy to reverse phenotyping of genetic findings, it would, e.g., also make sense for biomarker research in PD to assign a phenotype to biological signals instead of assigning biological signals to a phenotype (Sturchio et al. 2020).

Therefore, the multidimensional analysis of combined “dry” (clinical data including genetic information, artificial intelligence, imaging data including MRI, nuclear medicine, and ultrasound) and “wet” (biochemical or metabolic parameters in serum, urine, stool, and tissue samples) biomarker may provide sufficient information to facilitate accurate diagnosis and treatment in PD.

## A clinician’s view on PD biomarker

Over the past decades, many initiatives focused on research into clinically relevant diagnostic and prognostic biomarkers for Parkinson’s disease. Biomarkers are looked upon as excellent tools to screen for PD or PD-at risk-individuals. They may facilitate prodromal risk even before the onset of motor symptoms (Berg et al. 2013). However, to date, PD is mostly still diagnosed relatively late in the disease process, sometimes even years after the initial manifestation of motor symptoms. It is also the common belief that the application of an available disease-modifying treatment following the earlier diagnostic screening will reduce the currently abundant missing motivation of chronic neurodegenerative disease-at-risk individuals for a testing procedure (Müller 2017).

PD is mostly diagnosed, when approximately 60% of dopaminergic axons and 70% of nigral dopaminergic neurons are already gone. At that stage, frequently only transient motor symptoms occur (Chen 2010). The hypothesis is that a reliable and clinically relevant biomarker could enable an earlier implementation of, e.g., a disease-modifying therapy. A reliable biomarker should also enable the reflection of clinically relevant changes over the course of PD. An important step forward was to raise awareness for early non-motor signs based on initiatives for earlier detection of PD in the past years to characterize the so-called “prodromal” or “premotor” interval. However, heterogeneity of symptoms and the individually differing progression do not even allow a reliable classification of early signs and the various subtypes of the disease entity PD (Berg et al. 2013; Mahlknecht et al. 2015). Currently, no validated, reliable, specific, easy to apply biomarker for PD is yet available. A popular model of dopaminergic neuron degeneration, the Braak model, considers PD as α-synucleinopathy and sets Lewy body pathology as the essential lime light (Braak et al. 2003; Kingsbury et al. 2010; Halliday et al. 2012). However, recently failed anti-alpha-synuclein approaches with cinpanemab and similar results with prasinezumab leave doubts about aSyn as a therapeutic target in PD (Przuntek et al. 2004; Müller and Kohlhepp 2016; Müller et al. 2016; Jellinger 2019; Espay 2022). One could postulate that Lewy body pathology with aSyn pathology is not primarily responsible for nigral degeneration in PD (Muller et al. 2021; Espay, 2022) or that the enrichment of “pathologic” proteins as aSyn, tau or β-amyloid are not specific (Müller et al. 2021; Espay 2022). Concepts of purely aSyn-based biomarker in PD may fall short to address the entire pathophysiology of the disease.

On the other hand, there is no doubt that a certain genetic impact exists for some PD forms. More than 20 so-called “PD genes” are known. However, these genetic risk factors and mutations in familial PD forms, such as DJ-1, PINK-1, and UCHL-1, only account for approximately 10% of idiopathic PD patients. The penetration, the age of onset, and symptoms are highly variable. This was also shown in Glucocerebrosidase (GBA) mutation carriers (Thaler et al. 2020; Mullin et al. 2021; Greuel et al. 2020). Thus, these genetic mutations do not allow any conclusion on the progression of PD, which is essential from the patients’ view.

Another interesting approach to develop biomarkers for measuring the progression of PD was published recently. Here, a PD prediction algorithm compromised of ALDH1A1, LAMB2, UBE2K, SKP1A, and age was created by logistic regression for predicting progression to ≤ 70% Modified Schwab and England Activities of Daily Living within 3 years. Hoehn and Yahr Scale (HYS) had worsened from HY stage ≤ 2 to 3 (Rabey et al. 2020). This study demonstrates that a set of biomarker (instead of a single marker) could be more suitable to follow disease progression and should be equally considered for future biomarker development approaches.

Nevertheless, most PD patients suffer from sporadic PD forms (Emamzadeh and Surguchov 2018). There are environmental epigenetic influences, such as chronic toxin exposure or still unknown for instance viral or bacterial infections, all of which may serve as further still hypothetical causes for the onset of sporadic PD forms. Therefore, the concept of a more or less singular molecular pathologic biomarker approach is misleading, in particular when it is based on a pathological finding (Müller et al. 2021).

## Conclusions

Recent research into Parkinson's biomarker has not been able to identify a biochemical biomarker that could be translated into clinical practice so far. Clinical history taking and clinical examination are still the most important tools in the assessment of the disease. Different diagnostic tools are used to confirm the clinical diagnosis or to rule out other diseases in the differential diagnosis. Blood-based biomarker tests for PD are currently evaluated in research settings if they can help to support the diagnosis or rule out other diseases with relative probabilities. We realize that probably due to the rather heterogeneous spectrum of Parkinson's disease, no biomarker will be able to describe the disease using a “black and white” differentiation, to predict its course or even to clearly assign subtypes. Multidimensional analyses will be necessary for this in large patient cohorts, which, in addition to blood-based biomarker, should also include cerebrospinal fluid, tissue and other non-biological markers such as novel digital movement profile markers. A comparison of patient cohorts characterized in the best possible and multidimensional manner with the highest quality has the greatest long-term potential to contribute reliable results and to identify valuable biomarker sets for PD.
